# The geriatric nutritional risk index is an effective tool to detect GLIM-defined malnutrition in rectal cancer patients

**DOI:** 10.3389/fnut.2022.1061944

**Published:** 2022-11-15

**Authors:** Xi-Yi Chen, Yi Lin, Shang-Yu Yin, Ya-Ting Shen, Xi-Cheng Zhang, Ke-Ke Chen, Chong-Jun Zhou, Chen-Guo Zheng

**Affiliations:** ^1^Department of Cardiovascular and Thoracic Surgery, The Second Affiliated Hospital and Yuying Children's Hospital of Wenzhou Medical University, Wenzhou, China; ^2^Department of Endocrinology, The Third Affiliated Hospital of Shanghai University, The Wenzhou Third Clinical Institute Affiliated to Wenzhou Medical University, Wenzhou People's Hospital, Wenzhou, China; ^3^Department of Anorectal Surgery, The Second Affiliated Hospital and Yuying Children's Hospital of Wenzhou Medical University, Wenzhou, China

**Keywords:** GLIM, GNRI, PNI, ALI, malnutrition, rectal cancer

## Abstract

**Background:**

This study aimed to investigate the value of the Geriatric Nutritional Risk Index (GNRI), prognostic nutritional index (PNI), and advanced lung cancer inflammation index (ALI) scores in detecting malnutrition in patients with rectal cancer; the Global Leadership Initiative on Malnutrition (GLIM) was used as the reference criterion.

**Materials and methods:**

This study included patients with rectal cancer who underwent proctectomy. GNRI, PNI, and ALI were calculated to detect the GLIM-defined malnutrition using the Receiver operating characteristic (ROC) curves. Univariate and multivariate logistic regression analyses were used to evaluate the association between the nutritional tools and postoperative complications. Kaplan-Meier survival curves, log-rank tests, and univariate and multivariate Cox regression analyses were used to clarify the relationship between nutritional tools and overall survival (OS).

**Results:**

This study enrolled 636 patients with rectal cancer. The GNRI demonstrated the highest sensitivity (77.8%), pretty specificity (69.0%), and the largest AUC (0.734). The GNRI showed good property in predicting major postoperative complications. All three nutritional tools were independent predictors of OS.

**Conclusion:**

The GNRI can be used as a promising alternative to the GLIM and is optimal in perioperative management of patients with rectal cancer.

## Introduction

The third most common form of cancer is colorectal cancer (CRC), but the CRC-related mortality rate ranks second. In 2020, an estimated 1.9 million cases and 935,000 deaths will be attributed to colorectal cancer (including anal cancer), representing approximately one in 10 cancer cases and deaths (1). Patients with cancer often experience malnutrition, which is related with increased postoperative complications and mortality (2, 3). Thus, the nutritional status of patients with cancer should be assessed, and nutritional interventions should be provided as necessary in the perioperative period.

Many approaches have been used to screen and assess malnutrition. Additionally, quantitative nutritional tools have been developed to predict adverse outcomes. The geriatric nutritional risk index (GNRI) is an easy screening nutritional tool that combines serum albumin levels with ideal body weight to assess nutritional risk (4). The GNRI is related with poor prognosis in various malignancies and can be applied not only in elderly patients but also in young patients (5). The prognostic nutritional index (PNI), based on total lymphocyte counts and serum albumin levels, has been shown to be a prognostic indicator in many types of malignancies (6). The advanced lung cancer inflammation index (ALI), which is composed of serum albumin levels, neutrophil-lymphocyte ratio (NLR) and body mass index (BMI), is related with the poor outcomes in patients with different types of cancer (7–9). Based on the routine examination of biochemical and anthropometric measurements, all quantitative and objective nutritional tools facilitate the simplification of nutritional assessment and dynamic surveillance.

Despite the fact that malnutrition poses a major global health concern linked to an increased risk of morbidity, mortality, and costs, the clinical diagnostic criteria have not been universally agreed upon. To find an approach to secure broad global acceptance, the Global Leadership Initiative on Malnutrition (GLIM) has established a new consensual criteria report to build universal criteria for malnutrition diagnosis (10). GLIM is a two-step model for risk screening and diagnostic assessment. Since its introduction, the GLIM has been validated in a variety of diseases, including cancer, chronic liver disease, chronic kidney disease, and heart failure (11–14).

Quantitative nutritional tools have not been validated with the standard malnutrition diagnosis criteria as a reference for patients with rectal cancer. Therefore, we aimed to investigate the value of the GNRI, PNI, and ALI scores in detecting malnutrition using the GLIM as a reference criterion in patients with rectal cancer.

## Materials and methods

### Patients

This study included patients with rectal cancer who underwent proctectomy between January 2013 and April 2019 at the Anorectal Surgery Department of the Second Affiliated Hospital and Yuying Children's Hospital of Wenzhou Medical University. Inclusion criteria included the following: (1) age ≥ 18 years, (2) American Society of Anesthesiologists (ASA) grade ≤ III, and (3) available preoperative abdominal CT scans. Patients with metastatic cancer were excluded from this study. The data collection protocol for this study was approved by the Ethics Committee of the Second Affiliated Hospital and Yuying Children's Hospital of Wenzhou Medical University (LCKY2020–209). Informed consent was obtained from all participants.

### Data collection

Data was collected on the following parameters: (1) general features, including age, gender, height, weight, BMI, Charlson comorbidity index (CCI) score, ASA grade, and previous abdominal surgery; (2) laboratory features, including hemoglobin, albumin, neutrophil, lymphocyte, and NLR; (3) clinicopathological features, including tumor size, tumor location, tumor differentiation, tumor stage, node stage, and pathological tumor node metastasis (TNM); and (4) postoperative short-term and long-term outcomes, including postoperative major complications [major complications classified as Clavien–Dindo classification grade ≥ II. Complications of the highest grade were recorded when more than one type of complication occurred (15)] and mortality.

### Assessment of skeletal muscle index

Using specialized imaging software (INFINITT Healthcare Co, Ltd), preoperative abdominal CT images at the third lumbar vertebra (L3) level were obtained to determine skeletal muscle mass. Muscle tissues were identified using a Hounsfield unit (HU) threshold ranging from −29 to 150. Skeletal muscle index (SMI) was calculated as the cross-sectional area of the skeletal muscle mass divided by the square of the height (m). SMI cut off values were determined by our previous study (16).

### Nutritional assessment

GLIM is a two-step model for diagnosing malnutrition. The first step is to perform malnutrition risk screening to identify at-risk individuals. In this study, we used the Nutritional Risk Screening 2002 (NRS 2002). NRS 2002 ≥ 3 was considered to at risk of malnutrition. The second step requires at least one of the three phenotypic criteria [non-volitional weight loss, low BMI and reduced muscle mass] and one of the two etiologic criteria (reduced food intake or assimilation and disease burden/inflammation) for the diagnosis of malnutrition (10). The definition of non-volitional weight loss is exceeding 5% within 6 months or more than 10% beyond 6 months. Low BMI was defined as BMI <18.5 kg/m^2^ if patients aged ≥ 70 years, or BMI <20 kg/m^2^ if patients aged <70 years. Reduced muscle mass was defined as low SMI. Malnutrition was diagnosed based on the phenotypic criteria in this study, because one of the etiologic criteria (disease burden) had already been met.

GNRI was calculated as follows: GNRI = 1.489 × albumin (g/L) + 41.7 × present body weight/ideal body weight (the ideal body weight was calculated using Lorentz equations) (4). PNI formula was as follows: PNI = albumin (g/L) + 5 × total lymphocyte count (10^9^/L) (17). ALI was calculated using the following formula: ALI = BMI × albumin (g/dL) / NLR (9). According to Youden's index, a GNRI <98, PNI <45.5, or ALI <40 were defined as malnutrition.

### Follow-up

Follow-up with patients *via* telephone or outpatient visits was regularly conducted from enrollment until death, or until the end of the study in August 2022, or for more than 8 years. Patients were followed up 1 month after surgery, every 3 months for 2 years, and every 6 months thereafter. From the date of surgery until the date of death, overall survival (OS) was calculated.

### Statistical analysis

In continuous variables, mean and standard deviation (SD) or median and interquartile range (IQR) are shown. The categorical variable is presented as number and proportion. The optimal cutoff thresholds for the GNRI, PNI, and ALI are determined by receiver operating characteristic (ROC) curves with Youden's index correction. Univariate and multivariate logistic regression analyses are preformed to evaluate the relationship between the nutritional tools and postoperative complications. Kaplan-Meier survival curves, log-rank tests, and univariate and multivariate Cox regression analyses are used to clarify the association between nutritional tools and OS. Multivariate analysis is conducted on factors with *P* < 0.10 in the univariate analysis. Statistics assume significance when both sides of the *P*-value are lower than 0.05. The data were analyzed using SPSS version 26.0 and R software (version 4.2.1, https://cran.r-project.org).

## Results

This study enrolled 636 patients with rectal cancer. As shown in Table 1, the median age was 65 years, median height was 1.64 m, median weight was 60.99 kg, mean SMI was 42.57 cm^2^/m^2^, and median BMI was 22.41 kg/m^2^; furthermore, there were 385 (60.5%) male patients, and 200 (31.4) patients with CCI ≥1; the median tumor size was 4.0 cm, with 135 (21.2%) cases of lower location, and 82 (12.9%) cases of poor differentiation. There were 175 (27.5%) patients with TNM stage 0/I, 192 (30.2%) with stage II, and 269 (42.3%) with stage III. 158 (24.8%) patients were GLIM-defined malnutrition, and the malnutrition prevalence rates of GNRI, PNI, and ALI were 42.6, 31.0, and 46.9%, respectively.

**Table 1:** The patients' clinical characteristics.

**Characteristics**	**Overall (*n* = 636)**
**General feature**	
Age, median (IQR), years	65 (17)
<65	305 (48.0)
≥65	331 (52.0)
**Gender**	
Male	385 (60.5)
Female	251 (39.5)
Height, median (IQR), m	1.64 (0.08)
Weight, median (IQR), kg	60.99 (10.22)
SMI, mean (SD), cm^2^/m^2^	42.57 (8.49)
BMI, median (IQR), kg/m^2^	22.41 (4.07)
<18.5	72 (11.3)
18.5–23.9	369 (58.0)
≥24	195 (30.7)
**Charlson comorbidity index**	
0	436 (68.6)
≥1	200 (31.4)
**ASA grade**	
I	64 (10.1)
II	469 (73.7)
III	103 (16.2)
**Previous abdominal surgery**	
No	578 (90.9)
Yes	58 (9.1)
**Laboratory feature**	
Hemoglobin, median (IQR), g/L	130 (21)
Albumin, median (IQR), g/L	39.1 (5.4)
Neutrophil, median (IQR), 10^9^/L	3.69 (1.61)
Lymphocyte, median (IQR), 10^9^/L	1.74 (0.73)
Neutrophils/lymphocytes ratio, median (IQR)	2.12 (1.34)
**Clinicopathological feature**	
Tumor size, median (IQR), cm	4.0 (2.0)
**Tumor location**	
Upper	501 (78.8)
Lower	135 (21.2)
**Tumor differentiation**	
Well differentiated	554 (87.1)
Poorly differentiated	82 (12.9)
**Tumor stage**	
Tis, T1	58 (9.1)
T2	158 (24.8)
T3	353 (55.5)
T4	67 (10.5)
**Node stage**	
N0	370 (58.2)
N1	162 (25.5)
N2	104 (16.3)
**TNM stage**	
I, Tis	175 (27.5)
II	192 (30.2)
III	269 (42.3)
**Nutrition-related feature**	43.22 (8.58)
**NRS-2002**	
No nutritional risk	450 (70.8)
Nutritional risk	186 (29.2)
**Phenotypic criteria**	
Weight loss	54 (8.5)
Low BMI	99 (15.6)
Low skeletal muscle index	192 (30.2)
GLIM	114 (11.4)
Normal	478 (75.2)
Malnutrition	158 (24.8)
**GNRI**	
Normal	365 (57.4)
Malnutrition	271 (42.6)
**PNI**	
Normal	439 (69.0)
Malnutrition	197 (31.0)
**ALI**	
Normal	338 (53.1)
Malnutrition	298 (46.9)

Values are shown as number (%) unless otherwise indicated. IQR, interquartile range; SD, standard deviation; SMI, skeletal muscle index; BMI, body mass index; ASA, American Society of Anesthesiologists; TNM, tumor node metastasis; GLIM, Global Leadership Initiative on Malnutrition; GNRI, geriatric nutritional risk index; PNI, prognostic nutritional index; ALI, advanced lung cancer inflammation index.

**Figure 1:**
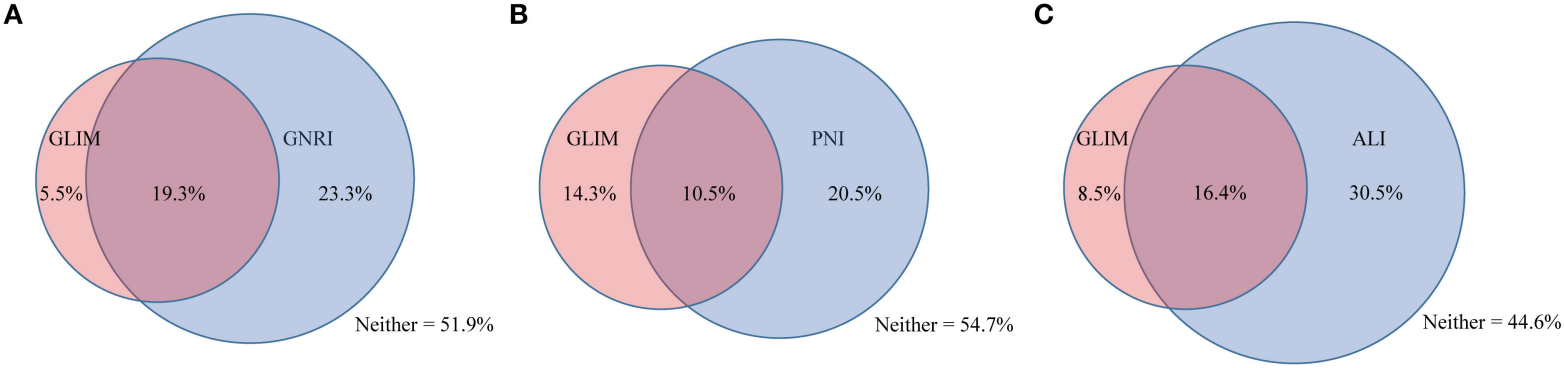
The relationship between GLIM and other nutritional tools. The relationship between GLIM and **(A)** GNRI, **(B)** PNI, **(C)** ALI. GLIM, Global Leadership Initiative on Malnutrition; GNRI, geriatric nutritional risk index; PNI, prognostic nutritional index; ALI, advanced lung cancer inflammation index.

Figure 1 shows the relationship between the nutritional tools and GLIM-defined malnutrition. Of the 24.8% of the cohort with GLIM-defined malnutrition, 19.3% were categorized as malnutrition by the GNRI, 10.5% were categorized as malnutrition by the PNI, and 16.4% were categorized as malnutrition by the ALI. A cross-tabulation of the nutritional tools and GLIM-defined malnutrition results is provided in Table 2.

**Table 2:** Cross tabulation of the results of nutritional tools and GLIM.

**Nutrition screening tool**	**GLIM-malnutrition**	**Normal**
**GNRI**
Score <98 (malnutrition)	123 (19.3)	148 (23.3)
Score ≥ 98 (normal)	35 (5.5)	330 (51.9)
**PNI**
Score <45.5 (malnutrition)	67 (10.5)	130 (20.5)
Score ≥ 45.5 (normal)	91 (14.3)	348 (54.7)
**ALI**
Score <400 (malnourished)	104 (16.4)	194 (30.5)
Score ≥ 400 (normal)	54 (8.5)	284 (44.6)

Values are shown as number (%). GLIM, Global Leadership Initiative on Malnutrition; GNRI, geriatric nutritional risk index; PNI, prognostic nutritional index; ALI, advanced lung cancer inflammation index.

Table 3 illustrates the sensitivity, specificity, positive predictive value, negative predictive value, positive likelihood ratio, negative likelihood ratio, and area under the curve (AUC) of the nutritional tools for identifying GLIM-defined malnutrition. The GNRI demonstrated the highest sensitivity (77.8%), pretty specificity (69.0%), and the largest AUC (0.734).

**Table 3:** Statistical evaluations of the nutritional tools compared with GLIM criteria for the diagnosis of malnutrition.

	**GNRI**	**PNI**	**ALI**
Sensitivity (%)	77.8	42.4	65.8
Specificity (%)	69.0	72.8	59.4
Positive predictive value (%)	45.4	34.0	34.9
Negative predictive value (%)	90.4	79.3	84.0
Positive likelihood ratio	2.5	1.6	1.6
Negative likelihood ratio	0.3	0.8	0.6
AUC	0.734	0.576	0.626

GLIM, Global Leadership Initiative on Malnutrition; GNRI, geriatric nutritional risk index; PNI, prognostic nutritional index; ALI, advanced lung cancer inflammation index. AUC, area under the curve.

As shown in Table 4, GLIM [odds ratio (OR): 1.735, 95% confidence interval (CI): 1.165–2.585; *P* = 0.007] and GNRI (OR: 1.647, 95% CI: 1.143–2.373; *P* = 0.007) were associated with postoperative complications in the univariate analysis. In the subsequent multivariate analysis, GLIM (OR: 1.865, 95% CI: 1.243–2.797; *P* = 0.003) and GNRI (OR: 1.669, 95% CI: 1.154–2.415; *P* = 0.007) were still associated with postoperative complications. Details of postoperative complications are shown in Supplementary Table 1.

**Table 4:** Univariate and multivariate logistic regression analysis of the association between the nutritional tools and postoperative complications.

	**Univariate analysis**	**Multivariate analysis^a^**
**Tools**	**HR (95% CI) *P***	**HR (95% CI) *P***
**GLIM**
Normal	Reference	Reference
Malnutrition	1.735 (1.165–2.585) 0.007*	1.865 (1.243–2.797) 0.003*
**GNRI**
Normal	Reference	Reference
Malnutrition	1.647 (1.143–2.373) 0.007*	1.669 (1.154–2.415) 0.007*
**PNI**
Normal	Reference	
Malnutrition	1.096 (0.743–1.617) 0.645	
**ALI**
Normal	Reference	
Malnutrition	1.403 (0.975–2.018) 0.068	

^*^Statistically significant (P < 0.05). GLIM, Global Leadership Initiative on Malnutrition; GNRI, geriatric nutritional risk index; PNI, prognostic nutritional index; ALI, advanced lung cancer inflammation index. ^*a*^Adjusted by age, gender, Charlson Comorbidity Index, ASA grade, previous abdominal surgery, tumor size, tumor location, tumor differentiation, TNM stage.

**Figure 2:**
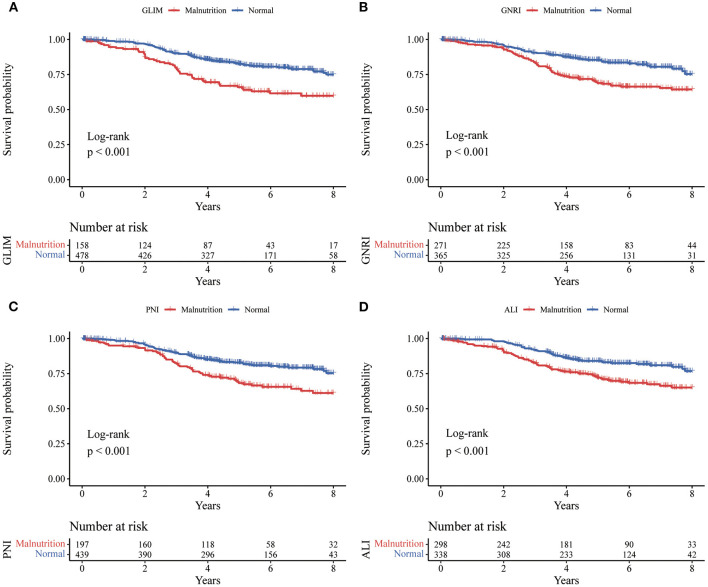
Kaplan-Meier curves for overall survival by the category of each tool in rectal cancer. Kaplan-Meier curves **(A)** for the GLIM, **(B)** for the GNRI, **(C)** for the PNI, and **(D)** for the ALI. GLIM, Global Leadership Initiative on Malnutrition; GNRI, geriatric nutritional risk index; PNI, prognostic nutritional index; ALI, advanced lung cancer inflammation index.

There were 135 deaths (21.2%) during follow-up. The median follow-up time was 4.94 years (IQR: 3.38–6.70). Figure 2 showed the Kaplan-Meier curves for overall survival by the category of each tool in rectal cancer. As shown in Table 5, GLIM (OR: 2.129, 95% CI: 1.542–2.872; *P* < 0.001), GNRI (OR: 1.975, 95% CI: 1.404–2.778; *P* < 0.001), PNI (OR: 1.871, 95% CI: 1.330–2.631; *P* < 0.001), and (OR: 1.862, 95% CI: 1.321–2.625; *P* < 0.001) were associated with worse OS. Considering the confounding factors in the multivariate analysis, GLIM (OR: 1.650, 95% CI: 1.147–2.375; *P* = 0.007), GNRI (OR: 1.478, 95% CI: 1.037–2.107; *P* = 0.031), PNI (OR: 1.539, 95% CI: 1.082–2.189; *P* = 0.016), and ALI (OR: 1.620, 95% CI: 1.143–2.297; *P* = 0.007) were still associated with worse OS.

**Table 5:** Univariate and multivariate Cox regression analysis of the association between the nutritional tools and overall survival.

	**Univariate analysis**	**Multivariate analysis^a^**
**Tools**	**HR (95% CI) *P***	**HR (95% CI) *P***
**GLIM**
Normal	Reference	Reference
Malnutrition	2.129 (1.542–2.872) <0.001*	1.650 (1.147–2.375) 0.007*
**GNRI**
Normal	Reference	Reference
Malnutrition	1.975 (1.404–2.778) <0.001*	1.478 (1.037–2.107) 0.031*
**PNI**
Normal	Reference	Reference
Malnutrition	1.871 (1.330–2.631) <0.001*	1.539 (1.082–2.189) 0.016*
**ALI**
Normal	Reference	Reference
Malnutrition	1.862 (1.321–2.625) <0.001*	1.620 (1.143–2.297) 0.007*

^*^Statistically significant (P < 0.05). GLIM, Global Leadership Initiative on Malnutrition; GNRI, geriatric nutritional risk index; PNI, prognostic nutritional index; ALI, advanced lung cancer inflammation index. ^*a*^Adjusted by age, gender, BMI, Charlson Comorbidity Index, ASA grade, previous abdominal surgery, tumor size, tumor location, tumor differentiation, TNM stage.

## Discussion

To our knowledge, this is the first study to investigate three nutritional tools GNRI, PNI, and ALI in detecting GLIM-defined malnutrition in patients with rectal cancer. The GNRI demonstrated the highest sensitivity (77.8%), pretty specificity (69.0%), and the largest AUC (0.734). GNRI is associated with postoperative complications and OS. Furthermore, all three nutritional tools were independent predictors of OS. The GNRI performs optimally among three nutritional tools, and we anticipate that it will substitute for the GLIM in specific situations.

The prevalence of GLIM-defined malnutrition ranged widely from 11.9 to 87.9% (11). Different subgroups of patients and different combinations of criteria in the GLIM criteria can explain these variations. In this study, the prevalence of GLIM-defined malnutrition was 24.8%, and other nutritional tools classified 31.0–46.9% of patients with rectal cancer as malnourished. Recently, Song et al. (3) reported that the prevalence of GLIM-defined malnutrition was 23.6% in patients with colorectal cancer, which is similar to the prevalence of GLIM-defined malnutrition in this study. Many previous studies have demonstrated that malnutrition is both a short and long-term risk factor. Malnutrition is a risk factor for postoperative complications and mortality in various malignancies, because malnutrition can affect the progression and therapeutic responses of cancer (18–20). Malnutrition is estimated to be responsible for 10–20% of deaths in patients with cancer rather than the tumor itself (21). Therefore, it is essential to assess the nutritional status of patients with cancer.

Previous studies compare the malnutrition risk screening tools that identify whether patients “at risk” status, like the NRS-2002, Malnutrition Universal Screening Tool (MUST), Mini Nutritional Assessment Short Form (MNA-SF) Patient-generated Subjective Global Assessment (PG-SGA) with the GLIM criteria in patients with cancer (22, 23). However, we do not believe that this is appropriate. GLIM emphasizes that identifying “at risk” status using a validated screening tool is the first key step in evaluating nutritional status. However, Zhang et al. (22) diagnosed GLIM-defined malnutrition without a first-step malnutrition risk screening. Huang et al. (23) reported no clear indication of which nutritional risk screening tool was used. Henriksen et al. (24) showed that different numbers of patients were diagnosed with malnutrition when different screening tools were used during the first step of the GLIM process. Thus, we compared three quantitative nutritional tools using the GLIM criteria in patients with rectal cancer. During the current COVID-19 pandemic, it has become more difficult to conduct traditional nutritional assessments and interventions because of social segregation and recommendations for reducing close contact. Quantitative and objective nutritional tools facilitate simplification of nutritional assessments and dynamic surveillance. Therefore, it is important to validate these nutritional tools.

In the present study, the GNRI was in good agreement with the GLIM. This association may be explained by the factors that constitute the indices. The GNRI is composed of serum albumin, present body weight and ideal body weight. Serum albumin levels have traditionally been considered to reflect the nutritional status and protein reserves of a person (25). There is also a close relationship between serum albumin levels and systemic inflammation in patients with cancer. Inflammatory cytokine levels surge as cancer cells progress, resulting in the albumin synthesis suppression, degradation promotion, and capillary escape (26). Therefore, serum albumin as a supportive proxy measure of inflammation is one of the etiologic criteria of GLIM (10). As for the other factors of the GNRI, the parameter of present body weight/ideal body weight cannot reflect the body composition precisely, while it may describe skeletal muscle mass macroscopically (4). In this study, the prevalence of reduced mass index was 30.2%, which was the most predominant of the three phenotypic criteria. This may explain why the GNRI has high agreement with the GLIM. Consistent with previous literature (5), we found that a low GNRI was negatively associated with postoperative complications and OS.

The PNI includes only two laboratory indicators (serum albumin and lymphocytes), without any anthropometric measurements. Serum albumin is a reflection of nutritional status and inflammation. Similar to serum albumin, lymphocytes reflect not only nutritional status, but also systemic inflammation (27). Accordingly, poor agreement with the GLIM for identifying malnutrition may be reasonable. In the present study, we found that PNI was associated with OS, but not with postoperative complications.

ALI, consisting of BMI, albumin, and NLR, is a recently described new tool for evaluating the nutritional status of patients with tumors. The specific feature of this index is a comprehensive formula that evaluates both nutritional status and inflammation because covariates of both aspects are included. Although BMI is used as a traditional nutritional indicator is used in the etiologic criteria for GLIM, the prevalence of low BMI is 15.6%. Huang et al. (28) reported that the prevalence of GLIM-defined malnutrition cannot be neglected by 11.9% of patients with obesity who have cancer. These factors may have contributed to the poor agreement between ALI and GLIM. Yin et al. (9) reported that ALI is associated with postoperative surgical site infection. Unfortunately, we did not classify postoperative complications as infectious or non-infectious in this study. In line with previous evidence, our study demonstrated that ALI is an independent prognostic marker for OS in patients with cancer.

This study has some limitations that should be considered. Firstly, even though we successfully validated our internal results, we did not conduct an external validation. Second, the nutritional tools were evaluated only once on admission. Dynamic changes in nutritional tools, which may be a better predictor of worse outcomes, were not examined in our study. Third, despite our attempts to minimize confounding factors, the retrospective nature of our analysis posed a risk of selection bias. Finally, this was a retrospective study among Chinese patients with rectal cancer, which may not be applicable to other ethnic populations and regions. In the future, a multicenter prospective study in different populations is required to validate our findings.

## Conclusion

In conclusion, this study demonstrated the superiority of GNRI in identifying GLIM-defined malnutrition and predicting postoperative complications in patients with PNI, and ALI. Regardless of the nutritional tools used to assess the nutritional status of the patients with rectal cancer, the OS of patients with malnutrition was worse than that of patients without malnutrition. Therefore, nutritional assessments should be highlighted in the management of patients with rectal cancer. In particular, the GNRI can be used as a promising alternative to the GLIM in some special situations, such as the current COVID-19 pandemic.

## Data availability statement

The datasets presented in this article are not readily available because the data presented in this study are available on request from the corresponding authors. The data are not publicly available due to patients' privacy. Requests to access the datasets should be directed to C-GZ, zhengchenguo_80@163.com.

## Ethics statement

This study was approved by the Ethics Committee of the Second Affiliated Hospital and Yuying Children's Hospital of Wenzhou Medical University (LCKY2020–209). Informed consent was obtained from all participants.

## Author contributions

C-GZ and C-JZ designed and revised the study. S-YY, X-CZ, and Y-TS collected the data. X-YC and K-KC did the analysis and interpretation of data. X-YC and YL did the drafting of manuscript. All authors contributed to the article and approved the submitted version.

## Funding

This study was funded by the National Natural Science Foundation of China (Grant Number: 82274530).

## Conflict of interest

The authors declare that the research was conducted in the absence of any commercial or financial relationships that could be construed as a potential conflict of interest.

## Publisher's note

All claims expressed in this article are solely those of the authors and do not necessarily represent those of their affiliated organizations, or those of the publisher, the editors and the reviewers. Any product that may be evaluated in this article, or claim that may be made by its manufacturer, is not guaranteed or endorsed by the publisher.

## References

[1] Sung H Ferlay J Siegel RL Laversanne M Soerjomataram I Jemal A. Global cancer statistics 2020: GLOBOCAN estimates of incidence and mortality worldwide for 36 cancers in 185 countries. CA Cancer J Clin. (2021) 71:209–49. 10.3322/caac.21660 33538338

[2] Lee DU Fan GH Hastie DJ Addonizio EA Suh J Prakasam VN. The clinical impact of malnutrition on the postoperative outcomes of patients undergoing colorectal resection surgery for colon or rectal cancer: propensity score matched analysis of 2011-2017 US hospitals. Surg Oncol. (2021) 38:101587. 10.1016/j.suronc.2021.101587 33915485

[3] Song HN Wang WB Luo X Huang DD Ruan XJ Xing CG. Effect of GLIM-defined malnutrition on postoperative clinical outcomes in patients with colorectal cancer. Jpn J Clin Oncol. (2022) 52:466–74. 10.1093/jjco/hyab215 35062024

[4] Bouillanne O Morineau G Dupont C Coulombel I Vincent JP Nicolis I. Geriatric nutritional risk index: a new index for evaluating at-risk elderly medical patients. Am J Clin Nutr. (2005) 82:777–83. 10.1093/ajcn/82.4.777 16210706

[5] Lv GY An L Sun DW. Geriatric nutritional risk index predicts adverse outcomes in human malignancy: a meta-analysis. Dis Markers. (2019) 2019:4796598. 10.1155/2019/4796598 31827634 PMC6885788

[6] Yan L Nakamura T Casadei-Gardini A Bruixola G Huang YL Hu ZD. Long-term and short-term prognostic value of the prognostic nutritional index in cancer: a narrative review. Ann Transl Med. (2021) 9:1630. 10.21037/atm-21-4528 34926674 PMC8640913

[7] Kusunoki K Toiyama Y Okugawa Y Yamamoto A Omura Y Ohi M. Advanced lung cancer inflammation index predicts outcomes of patients with colorectal cancer after surgical resection. Dis Colon Rectum. (2020) 63:1242–50. 10.1097/DCR.0000000000001658 33216495

[8] Song M Zhang Q Song C Liu T Zhang X Ruan G. The advanced lung cancer inflammation index is the optimal inflammatory biomarker of overall survival in patients with lung cancer. J Cachexia Sarcopenia Muscle. (2022) 13:2504–14. 10.1002/jcsm.13032 35833264 PMC9530543

[9] Yin C Toiyama Y Okugawa Y Omura Y Kusunoki Y Kusunoki K. Clinical significance of advanced lung cancer inflammation index, a nutritional and inflammation index, in gastric cancer patients after surgical resection: a propensity score matching analysis. Clin Nutr. (2021) 40:1130–6. 10.1016/j.clnu.2020.07.018 32773141

[10] Cederholm T Jensen GL Correia M Gonzalez MC Fukushima R Higashiguchi T. GLIM criteria for the diagnosis of malnutrition - a consensus report from the global clinical nutrition community. J Cachexia Sarcopenia Muscle. (2019) 10:207–17. 10.1002/jcsm.12383 30920778 PMC6438340

[11] Xu J Jie Y Sun Y Gong D Fan Y. Association of Global Leadership Initiative on Malnutrition with survival outcomes in patients with cancer: a systematic review and meta-analysis. Clin Nutr. (2022) 41:1874–80. 10.1016/j.clnu.2022.07.007 35944293

[12] Hirose S Matsue Y Kamiya K Kagiyama N Hiki M Dotare T. Prevalence and prognostic implications of malnutrition as defined by GLIM criteria in elderly patients with heart failure. Clin Nutr. (2021) 40:4334–40. 10.1016/j.clnu.2021.01.014 33551220

[13] Dai LL Li WL Zheng DF Wang WH Xie HF Ma JW. Prevalence and management recommendations for disease-related malnutrition in chronic kidney disease patients with and without diabetes. Int J Endocrinol. (2022) 2022:4419486. 10.1155/2022/4419486 36060295 PMC9436607

[14] Miwa T Hanai T Nishimura K Unome S Maeda T Ogiso Y. Usefulness of the Global Leadership Initiative on Malnutrition criteria to predict sarcopenia and mortality in patients with chronic liver disease. Hepatol Res. (2022) 52:928–36. 10.1111/hepr.13816 35861232

[15] Dindo D Demartines N Clavien PA. Classification of surgical complications: a new proposal with evaluation in a cohort of 6336 patients and results of a survey. Ann Surg. (2004) 240:205–13. 10.1097/01.sla.0000133083.54934.ae 15273542 PMC1360123

[16] Zhuang CL Huang DD Pang WY Zhou CJ Wang SL Lou N. Sarcopenia is an independent predictor of severe postoperative complications and long-term survival after radical gastrectomy for gastric cancer: analysis from a large-scale cohort. Medicine. (2016) 95:e3164. 10.1097/MD.0000000000003164 27043677 PMC4998538

[17] Onodera T Goseki N Kosaki G. [Prognostic nutritional index in gastrointestinal surgery of malnourished cancer patients]. Nihon Geka Gakkai Zasshi. (1984) 85:1001–5. 6438478

[18] Mauricio SF Xiao J Prado CM Gonzalez MC Correia M. Different nutritional assessment tools as predictors of postoperative complications in patients undergoing colorectal cancer resection. Clin Nutr. (2018) 37:1505–11. 10.1016/j.clnu.2017.08.026 28918167

[19] Arends J Bachmann P Baracos V Barthelemy N Bertz H Bozzetti F. ESPEN guidelines on nutrition in cancer patients. Clin Nutr. (2017) 36:11–48. 10.1016/j.clnu.2016.07.015 27637832

[20] Heber D Li Z. Nutrition intervention in cancer. Med Clin North Am. (2016) 100:1329–40. 10.1016/j.mcna.2016.06.011 27745597

[21] Muscaritoli M Arends J Bachmann P Baracos V Barthelemy N Bertz H. ESPEN practical guideline: clinical nutrition in cancer. Clin Nutr. (2021) 40:2898–913. 10.1016/j.clnu.2021.02.005 33946039

[22] Zhang Z Wan Z Zhu Y Zhang L Zhang L Wan H. Prevalence of malnutrition comparing NRS2002, MUST, and PG-SGA with the GLIM criteria in adults with cancer: a multi-center study. Nutrition. (2021) 83:111072. 10.1016/j.nut.2020.111072 33360034

[23] Huang Y Chen Y Wei L Hu Y Huang L. Comparison of three malnutrition risk screening tools in identifying malnutrition according to global leadership initiative on malnutrition criteria in gastrointestinal cancer. Front Nutr. (2022) 9:959038. 10.3389/fnut.2022.959038 35990353 PMC9386177

[24] Henriksen C Paur I Pedersen A Kvaerner AS Raeder H Henriksen HB. Agreement between GLIM and PG-SGA for diagnosis of malnutrition depends on the screening tool used in GLIM. Clin Nutr. (2022) 41:329–36. 10.1016/j.clnu.2021.12.024 34999327

[25] Gupta R Ihmaidat H. Nutritional effects of oesophageal, gastric and pancreatic carcinoma. Eur J Surg Oncol. (2003) 29:634–43. 10.1016/S0748-7983(03)00124-0 14511609

[26] Ballmer PE. Causes and mechanisms of hypoalbuminaemia. Clin Nutr. (2001) 20:271–3. 10.1054/clnu.2001.0439 11407876

[27] Zitvogel L Pietrocola F Kroemer G. Nutrition, inflammation and cancer. Nat Immunol. (2017) 18:843–50. 10.1038/ni.3754 28722707

[28] Huang DD Wu GF Luo X Song HN Wang WB Liu NX. Value of muscle quality, strength and gait speed in supporting the predictive power of GLIM-defined malnutrition for postoperative outcomes in overweight patients with gastric cancer. Clin Nutr. (2021) 40:4201–8. 10.1016/j.clnu.2021.01.038 33583658

