# Supplementary material for: The geriatric nutritional risk index is an effective tool to detect GLIM-defined malnutrition in rectal cancer patients
Source: Front Nutr. 2022 Nov 15;9:1061944. doi: 10.3389/fnut.2022.1061944 (PMC9705966; doi:10.3389/fnut.2022.1061944)
Supplement: Supplementary file 1 [file Table_1.DOC]

**Supplementary Table 1.** Detail of the postoperative complications

|  | Total |
| --- | --- |
| Detail of complications |  |
| Intra-abdominal infection | 13 (2.0) |
| Wound infection | 25 (3.9) |
| Bleeding | 5 (0.8) |
| Anastomotic leakage | 8 (1.3) |
| Ileus | 9 (1.4) |
| Gastrointestinal dysfunction | 7 (1.1) |
| Urinary infection | 21 (3.3) |
| Neurogenic bladder | 3 (0.5) |
| Pneumonia | 8 (1.3) |
| Anemia | 12 (1.9) |
| Persistent hypoalbuminemia | 16 (2.5) |
| Cardiac complications | 5 (0.8) |
| Cerebral infarction | 2 (0.3) |
| Venous thrombosis | 6 (0.9) |
| Sepsis | 3 (0.5) |
| Hyperthermia | 6 (0.9) |
| Others | 15 (2.4) |
| Clavien-Dindo classification grade |  |
| I | 12 (1.9) |
| II | 128 (20.1) |
| III | 17 (2.7) |
| IV | 8 (1.3) |
| V | 1 (0.2) |
| ^a^Major complications | 154 (24.2) |
| ^b^Severe complications | 26 (4.0) |

Values in parentheses are percentages.

^a^Major complications were defined as any adverse event corresponding to Clavien–Dindo classification grade II or higher, occurring within 30 days of surgery. If a patient had more than one type of complication, the complication with the highest grade was recorded.

^b^Severe complications were defined as Clavien–Dindo classification grade III or higher.
